# Emissive Enhancement of the Singlet Oxygen Chemiluminescence Probe after Binding to Bovine Serum Albumin

**DOI:** 10.3390/molecules24132422

**Published:** 2019-07-01

**Authors:** Jon Miranda-Apodaca, Nir Hananya, Adrián Velázquez-Campoy, Doron Shabat, Juan B. Arellano

**Affiliations:** 1 Departamento de Estrés Abiótico, Instituto de Recursos Naturales y Agrobiología de Salamanca (IRNASA-CSIC), 37008 Salamanca, Spain; 2 School of Chemistry, Faculty of Exact Sciences, Tel Aviv University, Tel Aviv 69978, Israel; 3 Institute of Biocomputation and Physics of Complex Systems (BIFI), Joint Units IQFR-CSIC-BIFI, and GBsC-CSIC-BIFI, Universidad de Zaragoza, 50018 Zaragoza, Spain; 4 Department of Biochemistry and Molecular and Cell Biology, Universidad de Zaragoza, 50009 Zaragoza, Spain; 5 Aragon Institute for Health Research (IIS-Aragon), 50009 Zaragoza, Spain; 6 Biomedical Research Networking Center in Digestive and Hepatic Diseases (CIBERehd), 28029 Madrid, Spain; 7 Fundación ARAID, Government of Aragon, 50018 Zaragoza, Spain

**Keywords:** chemically initiated electron exchange process, chemiluminescence, isothermal titration calorimetry, Schaap’s dioxetanes, singlet oxygen, SOCL, bovine serum albumin

## Abstract

A chemiluminescence probe for singlet oxygen ^1^O_2_ (SOCL) was investigated in phosphate buffer saline (PBS), either in the absence of proteins or containing bovine serum albumin (BSA). In the protein-free PBS, the reactivity of SOCL for methylene blue (MB)-photosensitized ^1^O_2_ was found to be moderate or low. The reaction yield increased with temperature and/or concentration of dissolved molecular oxygen. Unexpectedly, the presence of BSA boosted both the emissive nature and the thermal stability of the phenoxy-dioxetane intermediate formed in the chemiexcitation pathway. Isothermal titration calorimetry showed that SOCL has a moderate binding affinity for BSA and that entropy forces drive the formation of the SOCL-BSA complex. A model with two identical and independent binding sites was used to fit the binding isotherm data. Co-operative binding was observed when MB was present. Local viscosity factors and/or conformational restrictions of the BSA-bound SOCL phenoxy-dioxetane were proposed to contribute to the formation of the highly emissive benzoate ester during the chemically initiated electron exchange luminescence (CIEEL) process. These results led us to conclude that hydrophobic interactions of the SOCL with proteins can modify the emissive nature of its phenoxy-dioxetane, which should be taken into account when using SOCL or its cell-penetrating peptide derivative in living cells.

## 1. Introduction

Singlet oxygen (^1^O_2_) is a non-radical type of reactive oxygen species (ROS), characterized by its extremely low phosphorescence quantum yield and its very short lifetime in water [1,2]. Analysis of the temporal profile of the phosphorescence emission of ^1^O_2_ is difficult in aqueous buffer [3], but it becomes a herculean task when its temporal profile is to be measured in a biological (or crowded) system, where ^1^O_2_ is efficiently de-activated by the surrounding milieu or avidly reacts with electron-enriched compounds in living cells, and its lifetime can become as short as a few hundred nanoseconds [4,5]. The temporal profile of ^1^O_2_ was reported in HeLa cells after exchanging the intra-cellular water with deuterium oxide [6] and also in photodynamic inactivation of bacteria at different cell densities [7]; although, in the latter case, it was concluded that the rise and decay of the phosphorescence emission of ^1^O_2_ could have different meanings, depending on the molecular oxygen concentration in the medium [7]. The difficulty in measuring phosphorescence emission of ^1^O_2_ at ~1270 nm in living cells has caused scientists to look for alternative methods for its indirect detection and localization. The current use of bio-compatible fluorescent (and EPR) probes, together with bio-imaging techniques, has been established as one of the most sensitive approaches [8,9,10,11,12,13].

**Scheme 1:**
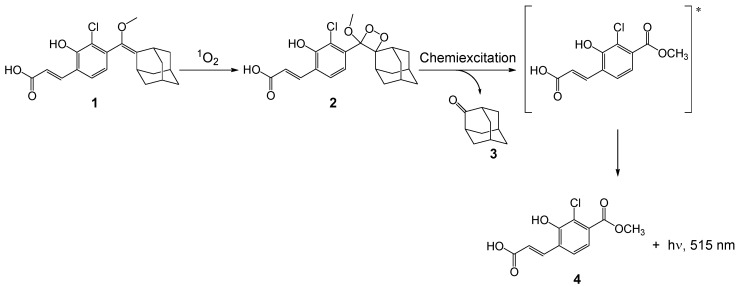
Structure of Singlet Oxygen Chemiluminescence probe (SOCL) and the reaction products of the chemiexcitation pathway upon the reaction of SOCL with ^1^O_2_. (**1**) 3-(4-((adamantan-2-ylidene)(methoxy)methyl)-3-chloro-2-hydroxyphenyl)acrylic acid (SOCL), (**2**) 3-(3-chloro-2-hydroxy-4-(4′-methoxyspiro[adamantane-2,3′-[1,2]dioxetan]-4′-yl)phenyl)acrylic acid (SOCL phenoxy-dioxetane), (**3**) adamantan-2-one, and (**4**) 3-(3-chloro-2-hydroxy-4-(methoxycarbonyl)phenyl)acrylic acid.

A new molecule, named Singlet Oxygen Chemiluminescence (SOCL), has been claimed to be the most effective chemiluminescence probe for real-time detection of ^1^O_2_ in living cells, with promising uses in photodynamic therapy-related applications [14]. SOCL is an enol-ether that reacts with ^1^O_2_ to form a Schaap’s dioxetane bearing a phenoxy substituent [15]. The SOCL 1,2-dioxetane spontaneously decomposes in aqueous media, through a chemically initiated electron exchange luminescence (CIEEL) process, to produce a highly emissive benzoate ester [14,16] (Scheme 1).

Schaap’s adamantylidene dioxetanes can be chemically modified to introduce appropriate electron withdrawing and/or protection groups in their phenoxy substituents, with the aim of evaluating different types of biological compounds in living cells [16,17,18,19,20,21]. SOCL-CPP, where CPP stands for cell penetrating peptide, was used to detect ^1^O_2_ production in living cells of HeLa after the covalent binding of a nona-arginine-glycine-cysteine peptide to its acrylic acid moiety [14]. The chemiluminescence features of SOCL and SOCL-CPP are advantageous when compared to those of other bio-compatible fluorescent probes, such as dansyl-2,2,5,5-tetramethyl-2,5-dihydro-1H-pyrrole (DanePy) or Singlet Oxygen Sensor Green (SOSG)—both probes have been widely used in the analysis of ^1^O_2_ production in living systems, for example, in plant cells or tissues [8]. One clear feature of SOCL is that light excitation is not required and, consequently, light scattering and background signals are minimal. DanePy requires UV excitation to image the loss of fluorescence emission after its reaction with ^1^O_2_. UV excitation might be damaging for the living cells or excite other macromolecules which emit fluorescence in the green/yellow region where DanePy emits. SOSG has a weak fluorescence, but it produces a green fluorescent endoperoxide derivative with high fluorescence quantum yield after its reaction with ^1^O_2_. The disadvantage of SOSG is that its endoperoxide derivative can also further react with molecular oxygen to artificially generate additional ^1^O_2_ [22]. Recently, the covalent link of SOSG to a polyacrylamide nanoparticle core has been shown to improve its stability and specificity for intra-cellular ^1^O_2_ [23].

Protein (or macromolecular) crowding in cell compartments affects both ^1^O_2_ diffusion and de-activation, due to changes in the solvent viscosity, protein dynamics, and physical/chemical interactions with key amino acid residues of proteins [24,25,26]. Proteins occupy about two-thirds of the dry matter in cells and are considered to be a major target for the damage initiated by ^1^O_2_ [24]. At present, a kinetics analysis of the chemical reactivity of SOCL (or SOCL-CPP) for ^1^O_2_ and the thermal decomposition of its phenoxy-dioxetane in living cells seems to be unrealistic. However, we can reach some primary conclusions about the interaction of SOCL with ^1^O_2_ in crowded systems if we make use of protein solutions that could resemble, to some extent, the protein concentration in cell compartments. Albumins from bovine serum (BSA) and human serum (HSA) are representative proteins, which have been used extensively in studies related to protein photo-oxidation and/or protein interactions with ^1^O_2_, photosensitizers, and photosensitizer carriers [27,28,29]. They are also of interest in ligand-protein interaction studies, due to their role in drug and nanoparticle delivery in vertebrates [30]. BSA and HSA have two binding sites for drugs: The drugs can bind to either site I (located in sub-domain IIA) through hydrophobic interactions or site II (located in sub-domain IIIA) through a combination of electrostatic, hydrogen bonding, and hydrophobic interactions.

In this report, kinetics and isothermal titration calorimetry (ITC) analyses show that the emissive nature of SOCL can be modified by temperature and oxygen concentration but is surprisingly enhanced after binding to BSA. The binding affinity of SOCL for BSA was attributed to entropy (hydrophobic) changes, which do not require a high degree of specificity.

## 2. Results

### 2.1. Temperature and Molecular Oxygen Effects on the Chemiluminescence Signal of the SOCL Phenoxy-Dioxetane

Methylene blue (MB), with a ^1^O_2_ quantum yield of approximately 0.52, was chosen as the photosensitizer in this study. This decision was based on the fact that MB is easily dissolved in phosphate buffer saline (PBS) pH 7.4, is photostable, and presents a flat rate of oxygen consumption during illumination (data not shown). Other considerations about MB will be given below, when BSA is used. MB was always present in the reaction mixture, with an absorbance of 0.15 at 663 nm (i.e., approximately 2 μM). After photosensitization, ^1^O_2_ deactivates in aqueous media with a lifetime of about 3.1–3.5 μs [2,31]. However, a steady state concentration of ^1^O_2_ can be reached, over time, in the reaction mixture if a continuous illumination is maintained. In the oxygen electrode, the chemical reaction of ^1^O_2_ with other molecular species can be followed indirectly by monitoring the consumption rate of molecular oxygen. When this is the case, one has to assume or test that other side-reactions consuming molecular oxygen are absent or negligible in the reaction mixture. In the case of SOCL, a high selectivity towards ^1^O_2_ (compared to other ROS) was demonstrated [14]. On this basis, no attempts were carried out to measure the formation of O_2_^•−^ or H_2_O_2_ in the oxygen electrode, under our experimental conditions.

**Figure 1:**
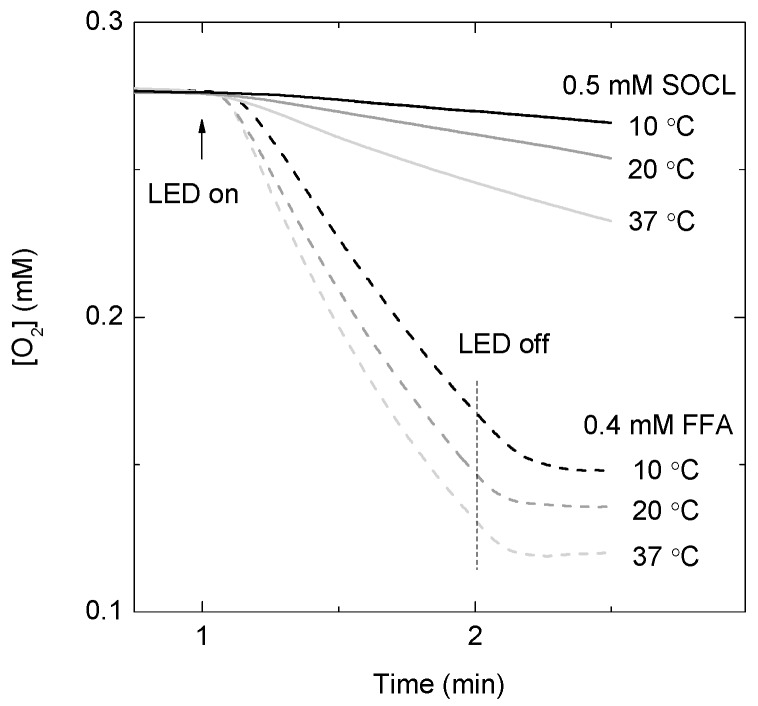
Molecular oxygen uptake in the oxygen electrode chamber containing 2 μM methylene blue as ^1^O_2_ photosensitizer and 0.5 mM Singlet Oxygen Chemiluminescence probe (SOCL), solid lines, or 0.4 mM furfuryl alcohol (FFA), dashed lines, as ^1^O_2_ quenchers in phosphate buffer saline pH 7.4 at three different temperatures. Samples were illuminated with a red light-emitting diode (LED) source with an intensity of 2 mE m^−2^ s^−1^ for 1 min or 2 min, depending whether FFA or SOCL were present in the reaction mixture, respectively. The photo-oxygenation reactions were performed under atmospheric pressure conditions.

Figure 1 shows the consumption rate of molecular oxygen in the presence of 0.5 mM SOCL at three different temperatures. The molecular ratio between SOCL and MB was approximately 250:1 in the experimental assays. The consumption rate of molecular oxygen at 37 °C was the highest (0.60 ± 0.03 μM s^−1^) and decreased with a decrease of temperature of the reaction mixture (Figure 1). At 4 °C, the rate of molecular oxygen consumption was very slow (data not shown) and, consequently, further experiments at temperatures below 10 °C were not undertaken in this study.

In order to estimate the order of magnitude of the bi-molecular reaction rate constant between SOCL and ^1^O_2_, the consumption rate of molecular oxygen was also measured in the presence of 0.4 mM furfuryl alcohol (FFA). FFA reacts selectively with ^1^O_2_, with a consumption ratio between FFA and molecular oxygen of 1:1 [32,33]. At room temperature and under low ionic strength, the bi-molecular reaction rate constant between FFA and ^1^O_2_ is approximately 1 × 10^8^ M^−1^ s^−1^. Taking into account both the equation for the temperature dependence of the bi-molecular reaction rate constant of FFA with ^1^O_2_ [32] and the oxygen consumption rates of FFA and SOCL in this study (Figure 1), the bi-molecular reaction rate constants of SOCL for ^1^O_2_ were estimated to be (8.0 ± 0.4) × 10^6^ M^−1^ s^−1^ at 20 °C and (2.0 ± 0.1) × 10^7^ M^−1^ s^−1^ at 37 °C.

**Figure 2:**
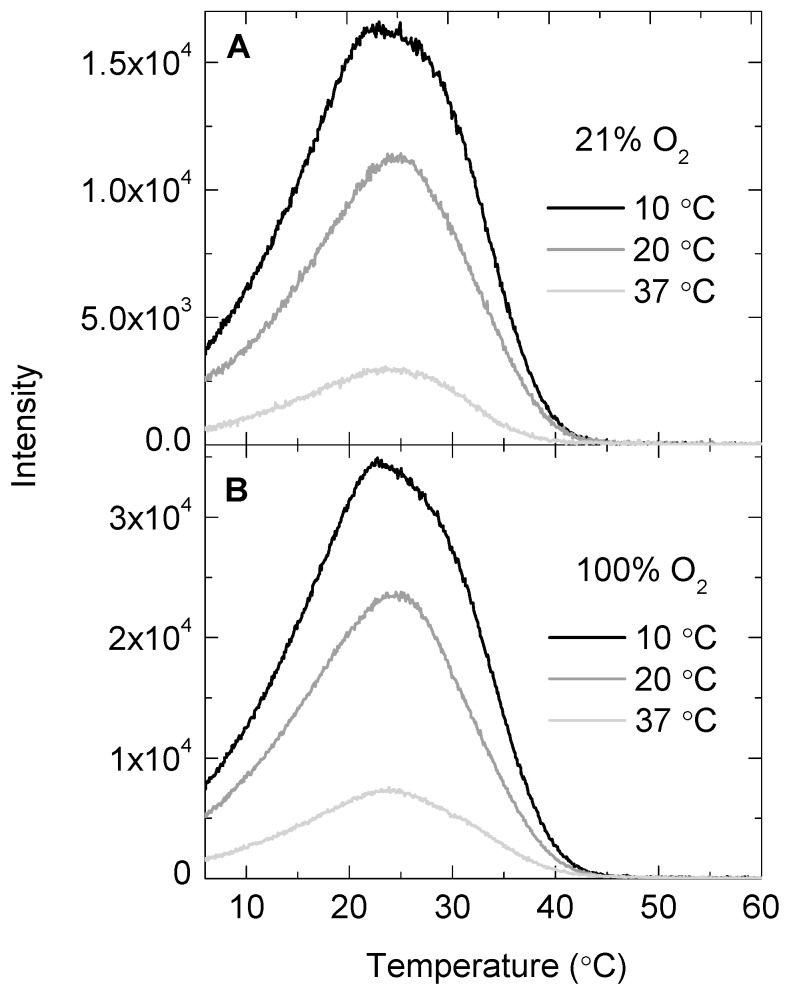
Temperature ramp of the chemiluminescence traces of the Singlet Oxygen Chemiluminescence (SOCL) phenoxy-dioxetane derivative after the photo-oxygenation of SOCL with ^1^O_2_ in phosphate buffer saline pH 7.4 at three different temperatures. The photo-oxygenation reactions were performed in equilibrium with an atmosphere of 21% O_2_ (**A**) or 100% O_2_ (**B**). Before monitoring the chemiluminescence traces, the reaction mixtures were set in the oxygen electrode chamber, where 2 μM methylene blue was present as ^1^O_2_ photosensitizer and 0.5 mM SOCL as ^1^O_2_ quencher. The reaction mixtures were illuminated with a red light-emitting diode source with an intensity of 2 mE m^−2^ s^−1^ for 2 min. Samples were taken from the oxygen electrode chamber after illumination and cooled down in a 1 cm fluorescence cuvette for 2 min to reach a temperature of 6 °C before the temperature ramp started. The rate of the temperature ramp was 5 °C min^−1^.

To demonstrate that the consumption rate of molecular oxygen indeed corresponded with the reaction of SOCL with ^1^O_2_, the chemiluminescence traces of the SOCL phenoxy-dioxetane were monitored at 515 nm with a spectrofluorometer. This dioxetane spontaneously decomposes through a chemiexcitation process in aqueous buffers at room temperature [14], but its decomposition was noticeably slower when the temperature was dropped to values around 4–6 °C (Figure 2A). The chemiluminescence traces exhibited an asymmetric bell-like shape, starting with a non-zero initial value at 6 °C. The intensity increased as the temperature rose up to 23–27 °C, where the chemiluminescence signal reached its maximum. After this point, the signal decreased until the SOCL phenoxy-dioxetane derivative completely decomposed. No chemiluminescence emission was observed beyond 50 °C when the temperature ramp was 5 °C min^−1^. The chemiluminescence signal of the SOCL phenoxy-dioxetane derivative could be further enhanced by a factor of two when 2 min illumination with a red light-emitting diode (LED) source was performed under an atmosphere of pure oxygen (Figure 2B). In this particular case, the oxygen consumption rates were not recorded, due to the limitations imposed by the technical design of the Chlorolab 2 system, which cannot be calibrated under an atmosphere of pure oxygen.

**Figure 3:**
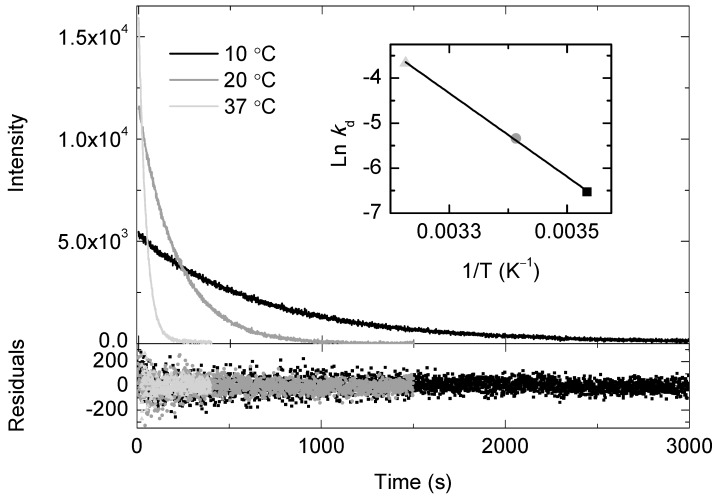
Time dependence of the chemiluminescence traces of the Singlet Oxygen Chemiluminescence (SOCL) phenoxy-dioxetane derivative after the photo-oxygenation of SOCL with ^1^O_2_ in phosphate buffer saline pH 7.4 at three different temperatures. Before monitoring the chemiluminescence traces, the reaction mixtures were set in the oxygen electrode chamber under atmospheric pressure conditions, where 2 μM methylene blue was present as ^1^O_2_ photosensitizer and 0.5 mM SOCL as ^1^O_2_ quencher. The reaction mixtures were illuminated with a red light-emitting diode source with an intensity of 2 mE m^−2^ s^−1^ for 2 min. Samples were taken from the oxygen electrode chamber after illumination and placed in a 1 cm fluorescence cuvette, which was pre-incubated at the temperature used in the oxygen electrode chamber. The inset shows the dependence of the decomposition rate constant of the SOCL phenoxy-dioxetane derivative on temperature, using the Arrhenius equation. The data was fitted to a mono-exponential equation, *I*_515nm_ = *α*_0_ + *α*_1_exp (−*t*/τ_1_). Also shown are the corresponding residuals for each temperature experiment. The standard errors for the decay rate constants are <5%.

The decomposition rate constant (*k*_d_) for the SOCL phenoxy-dioxetane derivative was also investigated in PBS, at pH 7.4 and for different temperatures (Figure 3). The initial chemiluminescence intensity was highest at 37 °C and the corresponding traces followed a mono-exponential decay, whose analysis yielded a value for *k*_d_ of (2.55 ± 0.08) × 10^−2^ s^−1^. The decomposition rate slowed down at lower temperatures and the values for *k*_d_ were (4.80 ± 0.05) × 10^−3^ s^−1^ at 20 °C and (1.45 ± 0.05) × 10^−3^ s^−1^ at 10 °C, respectively. Kinetics analysis of the chemiluminescence traces of the SOCL phenoxy-dioxetane derivative was also performed, at the selected temperatures, under an atmosphere of pure oxygen (Figure S1). In this latter case, the values of the initial intensities were higher, but the mono-exponential decay rates were not perturbed by the presence of a higher concentration of dissolved molecular oxygen. The values for *k*_d_ were the same as those values determined under air atmosphere, within experimental error. The analysis of the temperature dependence of *k*_d_ was performed using the Arrhenius equation. The insets of Figure 3 and Figure S1 show a linear relationship between Ln*k*_d_ versus T^−1^ in the temperature range between 10–37 °C. For both Arrhenius plots, a value of (76.9 ± 0.4) kJ mol^−1^ was determined for the activation energy of the thermal decomposition of the SOCL phenoxy-dioxetane derivative in PBS (pH 7.4).

### 2.2. BSA Effect on the Chemiluminescence Signal of the SOCL Phenoxy-Dioxetane

**Figure 4:**
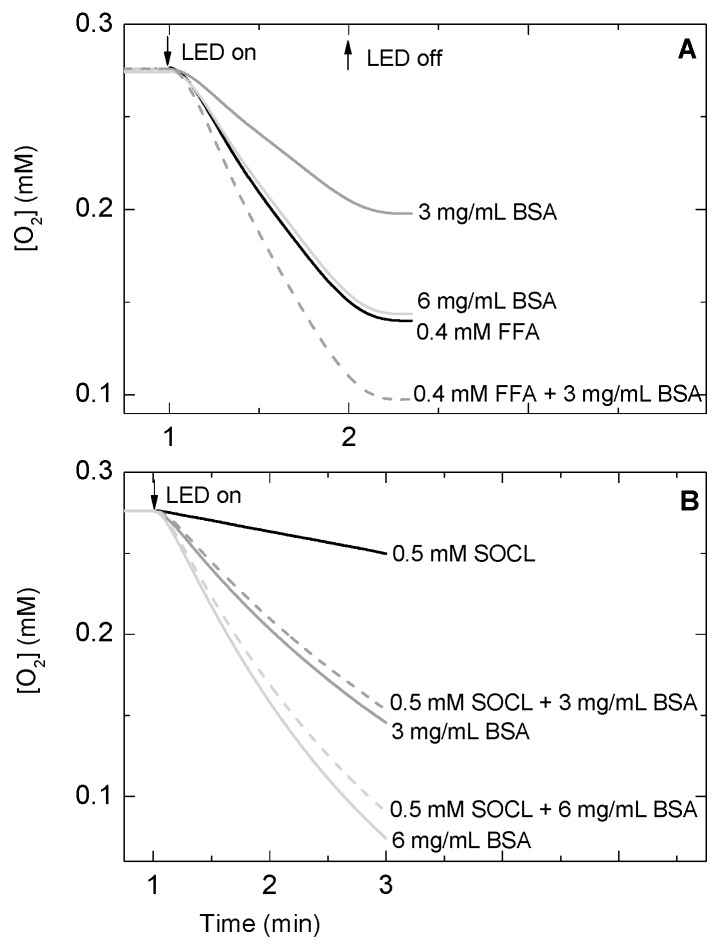
Effect of bovine serum albumin (BSA) concentration on the molecular oxygen uptake of several reaction mixtures containing 2 μM methylene blue as ^1^O_2_ photosensitizer and furfuryl alcohol (FFA) at 0 or 0.4 mM (**A**) and Singlet Oxygen Chemiluminescence probe (SOCL) at 0 or 0.5 mM (**B**) as ^1^O_2_ quenchers in phosphate buffer saline pH 7.4 at 20 °C. Samples were illuminated with a red light emitting diode (LED) source with an intensity of 2 mE m^−2^ s^−1^ for 1 min (**A**) or 2 min (**B**). The photo-oxygenation reactions were performed under atmospheric pressure conditions.

A homogenous protein solution using BSA was prepared, with the aim of studying the chemiluminescence signal of the SOCL phenoxy-dioxetane derivative in an environment that resembles the protein crowding of cell compartments. The oxygen consumption experiments started with the addition of BSA to PBS at pH 7.4 containing approximately 2 μM MB. The molecular ratio between BSA and MB ranged between 22:1 and 45:1. As shown in Figure 4, illumination of the reaction mixture containing BSA and MB triggered molecular oxygen consumption, with a rate that depended on BSA concentration. A concentration of 3 mg mL^−1^ (or 45 μM) of BSA gave an oxygen consumption rate that was about half that of 0.4 mM FFA, a protein concentration that was simply 0.7%–1% of the total protein concentration of some cell organelles. For example, plant chloroplasts—where ^1^O_2_ is also produced by photosystem II—contains a protein concentration in the stroma of about 300–400 mg mL^−1^ [34,35]. When the concentration of BSA was doubled, the oxygen consumption rate also increased, although the increase was not completely linear. MB (or MB/TiO_2_) is known to bind predominantly to BSA or HSA in one single site. MB binds to BSA or HSA within the sub-domain IIA with a moderate association constant, whose values range from 2.4 × 10^3^ M^−1^ [36] to 7.6 × 10^4^ M^−1^ [37] for BSA and from 4.0 × 10^4^ M^−1^ [38] to 4.3 × 10^4^ M^−1^ [39] for HSA. Based on the former association constants, the BSA-bound MB was calculated to increase by approximately 8–10% when the BSA concentration went from 45 μM to 90 μM in the reaction mixture. The change in the ratio between free MB and BSA-bound MB could, thus, be responsible for both a more local production of ^1^O_2_ in the solvent surrounding the BSA and a non-linear effect on the oxygen consumption rate. It has been previously reported that molecular oxygen efficiently quenched the triplet excited state of HSA-bound MB, and also that HSA did not modify the initial ^1^O_2_ phosphorescence intensity or its rise rate constant [28]. This indicates that ^1^O_2_ production is not affected by the binding of MB to HSA (and, presumably, to BSA), although its quenching can, in contrast, be differently perturbed by the local protein environment, which can either activate or inhibit the reaction of ^1^O_2_ with key amino acid residues of proteins [40].

When FFA was added to the reaction mixture containing BSA, the oxygen consumption rate increased, suggesting that both FFA and BSA competed for and reacted with ^1^O_2_ (Figure 4A). However, the addition of 0.5 mM SOCL to the reaction mixture containing BSA showed an effect on the molecular oxygen consumption rate that did not follow the trend observed for 0.4 mM FFA. In fact, the oxygen consumption rate of SOCL and BSA together was slightly slower than the one observed when BSA was the only ^1^O_2_ quencher in the reaction mixture. This was observed using either 3 mg mL^−1^ or 6 mg mL^−1^ BSA (Figure 4B).

**Figure 5:**
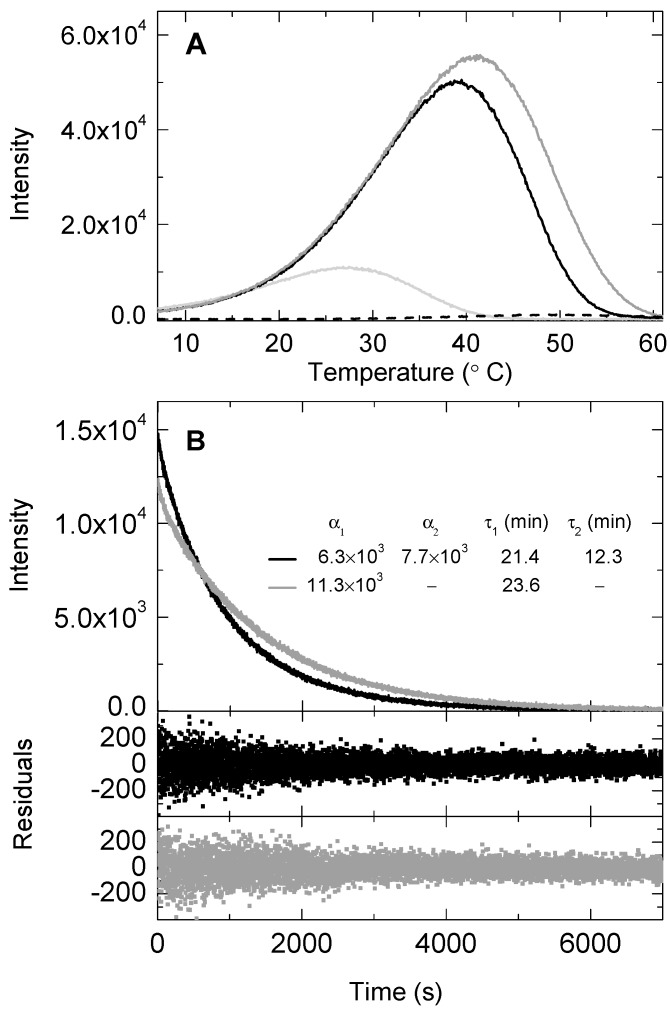
Temperature (**A**) and time dependence (**B**) of the chemiluminescence traces of the Singlet Oxygen Chemiluminescence (SOCL) phenoxy-dioxetane derivative after the photo-oxygenation reaction of SOCL with ^1^O_2_ in the presence of bovine serum albumin (0 mg mL^−1^, solid light grey line; 3 mg mL^−1^, solid black line; 6 mg mL^−1^, solid dark grey line; and 6 mg mL^−1^ without SOCL addition, dashed black line) in phosphate buffer saline pH 7.4. Before monitoring the chemiluminescence traces, the reaction mixtures were set in the oxygen electrode chamber under atmospheric pressure conditions, where 2 μM methylene blue was present as ^1^O_2_ photosensitizer and 0.5 mM SOCL as ^1^O_2_ quencher. The reaction mixtures were illuminated with a red light emitting diode source with an intensity of 2 mE m^−2^ s^−1^ for 2 min at 20 °C. For the temperature ramp experiments, samples were taken from the oxygen electrode chamber after illumination and cooled down in a 1 cm fluorescence cuvette for 2 min to reach a temperature of 6 °C before monitoring the chemiluminescence traces. For the time-based experiments, samples were taken from the oxygen electrode chamber after illumination and placed in a 1 cm fluorescence cuvette pre-incubated at 20 °C. The data was fitted to a bi-exponential equation, *I*_515nm_ = *α*_0_ + *α*_1_exp (−*t*/τ_1_) + *α*_2_exp (−*t*/τ_2_). Also shown are the corresponding residuals for each temperature experiment. The standard errors for the decay rate constants are <5%.

To unambiguously ascertain whether SOCL could compete with BSA for ^1^O_2_, the chemiluminescence of the SOCL phenoxy-dioxetane derivative was also examined in the presence of BSA after 2 min illumination in the oxygen electrode at 20 °C. Figure 5 shows an unexpected effect of BSA on the chemiluminescence traces. The area under the curve, remarkably, increased by a factor of 5–6 in the presence of BSA and its maximum was shifted to higher temperatures (Figure 5A). The chemiluminescence maximum and intensity of the SOCL phenoxy-dioxetane derivative depended on the BSA concentration. To exclude the possibility that the observation was intrinsically associated with a chemiluminescence signal coming from the photo-oxidative decomposition of amino acid residues of BSA, a temperature ramp was also carried out in a solution containing BSA and MB. The signal was of little significance when BSA was the only ^1^O_2_ quencher in the reaction mixture (Figure 5A).

The shift of the chemiluminescence maximum to higher temperatures in the presence of BSA suggested that the SOCL phenoxy-dioxetane derivative was formed and was, indeed, more thermostable. This hypothesis was reinforced when the time-dependence of the chemiluminescence traces of the SOCL phenoxy-dioxetane derivative was inspected. The decay rate was slower and required a bi-exponential fitting when BSA was at 3 mg mL^−1^ in the reaction mixture, providing evidence for two different populations of SOCL with a ratio for α_1_:α_2_ of 45:55 in the reaction mixture (Figure 5B). The pre-exponential ratio changed when the concentration of BSA increased, up to 6 mg mL^−1^. In this latter case, the fitting only required a mono-exponential equation, whose decay rate constant was rather similar to the lowest decay rate constant required for the bi-exponential fitting in the presence of 3 mg mL^−1^ of BSA.

### 2.3. SOCL and BSA Binding Energetics

**Figure 6:**
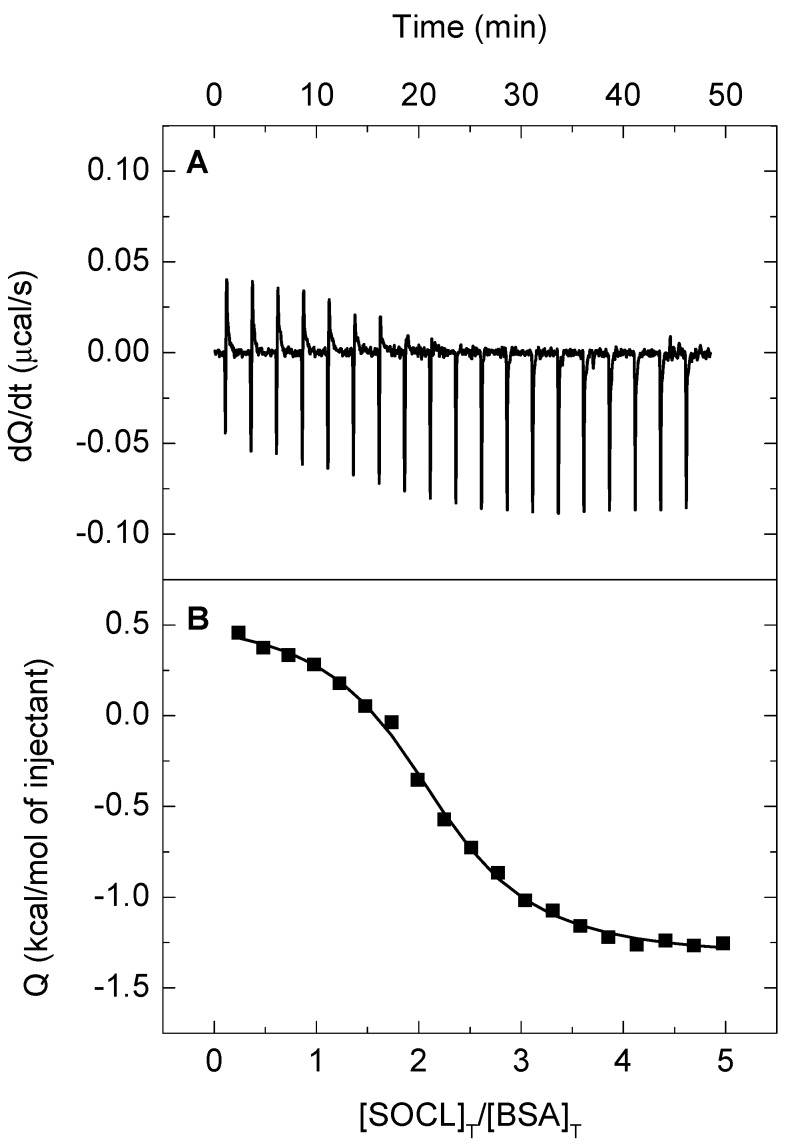
Calorimetric titration of bovine serum albumin (BSA) with Singlet Oxygen Chemiluminescence probe (SOCL) in phosphate buffer saline pH 7.4. The upper panel (**A**) shows the heat effects associated with the injection of SOCL (2 μL per injection of a 300 μM solution) into the calorimetric cell (200.7 μL) containing monomeric BSA at a concentration of 12.5 μM. The experiments were performed at 25 °C. The lower panel (**B**) shows the binding isotherm corresponding to the data in the upper panel and the best-fitted curve, considering a model for two identical and independent ligand binding sites in BSA (see the text for further details).

Figure 6A shows the thermogram (thermal power raw data as a function of time) of a calorimetric titration of SOCL into BSA. The background injection effect (i.e., the SOCL dilution and the contribution from mechanical mixing of solutions, as well as other possible unspecific effects) was exothermic and its contribution to the total heat observed after each injection was accounted for, through an adjustable parameter in the ITC analysis.

Figure 6B shows the binding isotherm (ligand-normalized integrated heats as a function of the molar ratio) data, along with a non-linear fitting that corresponded to the theoretical heat produced for the formation of a 2:1 complex between SOCL and BSA, where the two binding sites in the protein did not interact and had the same intrinsic affinity for the ligand (i.e., two identical and independent binding sites). The association constant had a value of (5.9 ± 0.9) × 10^5^ M^−1^ and the heat effect associated with the binding was endothermic (Δ*H*, 7.9 ± 1.2 kJ mol^−1^ at 25 °C). Table 1 summarizes the best-fitted values for the parameters.

## 3. Discussion

The photo-oxygenation of carbon-carbon double bonds to form 1,2-dioxetanes has been the focus of a considerable amount of research since the early 1970s, when the first stable 1,2-dioxetane from tetramethoxy ethylene was prepared in an irradiated organic solvent containing ^1^O_2_ photosensitizers of different natures [41]. Based upon this seminal study and the high selectivity of this oxidative reaction for ^1^O_2_, stable (as well as non-stable) 1,2-dioxetane precursors have been synthesized, with the aim of developing chemiluminescence detection methods for ^1^O_2_ in aqueous media [14,42]. SOCL and, particularly, SOCL-CPP were chemically designed to detect ^1^O_2_ in living organisms in real-time [14]. The substituents at the two *ortho* positions of its phenol were tailored to enhance the emissive nature of the SOCL phenoxy-dioxetane [14]. Here, we discuss how the emissive nature of the SOCL phenoxy-dioxetane is further enhanced after the entropy-driven binding of SOCL to BSA. We conclude that the hydrophobic interactions of SOCL with proteins should, thus, be taken into account when using SOCL or SOCL-CPP in living cells.

First, we analyzed the rate constant of the bi-molecular reaction between SOCL and ^1^O_2_ at different temperatures, with the aim of gaining a better knowledge of the reactivity of SOCL towards ^1^O_2_. The values for the bi-molecular reaction rate constant ranged (in order of magnitude) from 10^6^ M^−1^ s^−1^ at 20 °C to 10^7^ M^−1^ s^−1^ at 37 °C. The effect of SOCL on the triplet excited state of MB was not investigated. In fact, molecular collisions can de-activate the triplet excited state of MB and, thus, decrease the production of the SOCL phenoxy-dioxetane derivative in the reaction mixture. Although we were aware of this side-reaction, the values determined for the bi-molecular reaction rate constants between SOCL and ^1^O_2_ were in line with those reported for other adamantyl-substituted 1,2-dioxetanes [42]. This suggested that the de-activation of the triplet excited of MB by the ground state SOCL may indeed play a minor role in PBS (pH 7.4). In comparison with the bi-molecular reaction rate constant of FFA for ^1^O_2_ [32,33], the reactivity of SOCL for ^1^O_2_ was considered to be low and/or moderate (Figure 1). In spite of the low reactivity of SOCL for ^1^O_2_, particularly at low temperatures, it is worthwhile to note that the integrated chemiluminescence signal increased notably (Figure 3) and could even be multiplied by a factor of two when the bi-molecular reaction between SOCL and ^1^O_2_ was carried out under an atmosphere of pure oxygen (Figure S1). Solvent viscosity has been shown to perturb the chemiluminescence quantum yield of inter- and also intra-molecular CIEEL processes [43,44] and this could explain, at least partly, the chemiluminescence enhancement of the SOCL phenoxy-dioxetane at lower temperatures. The viscosity of water goes from 0.69 cP at 37 °C to 1.37 cP at 10 °C. Both temperature and oxygen concentration can be easily modified in test tube assays or cell cultures and, so, they can become part of chemical strategies to enhance the signal-to-noise ratio of the chemiluminescence signal of the SOCL phenoxy-dioxetane derivative.

While some Schaap’s adamantylidene dioxetanes can be thermally stable and have lifetimes of several years at room temperature, others 1,2-dioxetanes are quite unstable and do not require chemical or enzymatic triggers for chemiexcitation [15,45,46]. De-protonation of the phenol in phenoxy-substituted 1,2-dioxetanes modifies the Arrhenius activation energy for thermal decomposition, converting stable 1,2-dioxetanes into non-stable ones [15]. The SOCL phenoxy-dioxetane belongs to the latter class of 1,2-dioxetanes [14,16]. Over the entire temperature range used in this study, the decay rate of the SOCL phenoxy-dioxetane in PBS (pH 7.4) was first-order and the value for its Arrhenius activation energy explained its thermal decomposition behavior well (Figure 3 and Figure S1). Unexpectedly, we observed that the thermostability of the SOCL phenoxy-dioxetane increased and its chemiluminescence was boosted by a factor of 4–5 at room temperature simply with the addition of BSA to the solution (Figure 5).

The ITC analysis confirmed that SOCL bound to BSA, in two identical and independent sites, with a moderate association constant (Figure 6). After the photo-oxygenation reaction, the SOCL phenoxy-dioxetane was proposed to remain in the binding sites until spontaneous decomposition. This was inferred from the changes in the decomposition rate of the SOCL phenoxy-dioxetane, whose lifetime at 20 °C went from 3.5 min in the absence of BSA to 23.6 min at the highest concentration of BSA (Figure 5). The thermal decomposition rate followed a mono-exponential decay at 90 μM BSA, but a bi-exponential decay at 45 μM BSA. Although further kinetics analyses might be required to clarify this issue, we could, however, exclude that the decomposition reaction of the SOCL phenoxy-dioxetane took place in the bulk solvent when BSA was present. Based on the concentrations of the reactants and their respective association constants, and also assuming that SOCL and MB showed competitive binding for the sub-domain IIA of BSA, the concentrations at equilibrium (before illumination) of free MB and SOCL ought to be high enough to show evidence for the decomposition rate of free SOCL phenoxy-dioxetane. However, this was not the case when the fitting analysis was performed after illumination. When a third exponential decay was included in the kinetics analysis and fixed with a decay rate constant of (4.80 ± 0.05) × 10^−3^ s^−1^ at 20 °C, its pre-exponential was about 5% of the sum of pre-exponentials. This led us to propose both that the concentration of free MB was very small in solution when BSA was at high concentrations and, also, that SOCL and MB had to form a ternary complex together with BSA. Co-operative binding of two ligands to BSA or HSA has been previously reported [27,38]. Further evidence for the ternary complex in our study was the subtracting effect of SOCL on the overall oxygen consumption rate when BSA was present in solution. At this point, we suggest that either molecular collisions between molecular oxygen and the triplet excited state of MB were hindered in the ternary complex or, alternatively, there was static fluorescence quenching due to molecular contacts between the ground states of MB and SOCL. Although less efficient than other heavy halides [47], the aryl chloride of SOCL could play a role as a fluorescence quencher.

Regardless of the origin of the lower oxygen consumption in the reaction mixture when the ternary complex was formed, the SOCL phenoxy-dioxetane showed a boost in chemiluminescence and higher stability in the presence of BSA. The association constant between SOCL and BSA had a positive change in Δ*H* (Table 1). This implied that entropy changes and, consequently, hydrophobic interactions were responsible for the moderate binding affinity of SOCL for BSA. Conformational and solvation entropies are known to be the two main forces controlling the overall entropy changes in protein-ligand complexes [48,49,50]. The chemical structure of SOCL suggests that the molecule is relatively rigid (Scheme 1). The acrylic acid and non-polar adamantylidene group of SOCL cannot rotate freely around the conjugated double bond system. Therefore, the loss of conformational degrees of freedom of SOCL after its binding to BSA is probably low. In contrast, the solvation entropy is expected to increase after the expulsion of water molecules, at least, in great part due to the burial of the non-polar and bulky adamantylidene group of SOCL. So, the solvation entropy is suggested to be the main driving force for the interaction between SOCL and BSA. After the complex formation, the binding sites can also act as local viscosity factors.

Experimental evidence for the viscosity dependence of intra- and inter-molecular CIEEL processes has been reported in different solvent mixtures [43,44]. Recently, a concerted mechanism requiring a specific conformation for the functional groups in the back electron transfer was proposed, in order to explain the viscosity dependence of the intra-molecular CIEEL process [51]. After the photo-oxygenation reaction, the protein environment could play a key role in the CIEEL process, restricting conformational changes in the SOCL phenoxy-dioxetane. This means that molecular rigidification could, indeed, be the main cause for the emissive enhancement of the benzoate ester formed in the chemiexcitation pathway. Other protein-ligand interactions that could stabilize the (bi-radical) intermediate species during the CIEEL process cannot be discarded.

## 4. Material and Methods

### 4.1. Chemical Reagents and Stock Solutions

Synthesis of the SOCL probe, with a molecular mass of 374.86 g mol^−1^, was previously described, in detail, in reference [14]. MB, FFA, BSA, 10× concentrate PBS, and the other chemicals were purchased from Sigma-Aldrich. After ten-fold dilution, PBS had a pH of 7.4 at 25 °C. Stock solutions of SOCL at different concentrations were prepared by dissolving the dry powder of SOCL in dimethyl sulfoxide. Stock solutions of FFA were prepared in ethanol.

### 4.2. Oxygen Consumption Experiments

Oxygen consumption was monitored, as described in one of our previous studies [52] with some modifications. In brief, oxygen uptake by the ^1^O_2_ quencher FFA or SOCL was measured polarographically using a Chlorolab 2 system (Hansatech Instruments Ltd., Norfolk, England, UK). MB was used in this study to photosensitize ^1^O_2_. All the samples were incubated in the liquid-phase electrode chamber in the dark for 1 min before the red LED source was switched on. The light irradiance in the electrode chamber was 2 mE m^−2^ s^−1^. In each experiment, the red LED source was on for 1 or 2 min, depending on whether FFA or SOCL were present in the solution, respectively. The illumination period with SOCL was prolonged with the sole purpose of increasing the concentration of the SOCL phenoxy-dioxetane derivative in the reaction mixture. When needed, the solutions were saturated with pure molecular oxygen. In this latter case, the samples were incubated within the liquid-phase electrode chamber for 15 min under a stream of pure molecular oxygen while the red LED source was off. The stream of pure molecular oxygen was maintained during the illumination period. The oxygen electrode assay conditions when BSA was present in the reaction mixture were explained, in further detail, in Section 2.

### 4.3. Chemiluminescence Experiments

The chemiluminescence signal of the SOCL phenoxy-dioxetane derivative was measured in a PTI spectrofluorimeter Model QM-2000-4 (Photon Technology International Inc., Lawrenceville, NJ, USA). The source light from the Xe arc lamp was blocked with an excitation monochromator shutter, to avoid any interference during the chemiluminescence measurements. After 2 min of illumination in the Chlorolab 2 system, the reaction mixture was taken from the electrode chamber and loaded into a 1-cm fluorescence cuvette, which was pre-incubated to an initial temperature that depended on the type of measurement programmed in the spectrofluorimeter. The chemiluminescence signal of the SOCL phenoxy-dioxetane derivative was monitored at 515 nm while stirring the solution. Two types of chemiluminescence emission experiments were designed. In the first instance, temperature ramp experiments with a constant rate from 6 to 60 °C were designed, in order to monitor the temperature effect on the chemiluminescence signal of the SOCL phenoxy-dioxetane derivative. A rate of 5 °C min^−1^ was chosen to guarantee a homogenous increase in temperature inside the cuvette. A PE94 temperature controller (Linkam Scientific Instruments Ltd., Tadworth, England, UK) with an Eheim water circulator (Eheim GmbH & Co KG, Stuttgart, Germany) was used to follow the temperature rate. In the temperature ramp experiments, the 1 cm fluorescence cuvette was always pre-incubated in an ice bath to drop the temperature of the reaction mixture after the 2 min illumination in the oxygen electrode chamber. The initial temperature of the ramp was set at 6 °C, in order to avoid water condensation around the cuvette surface. In the second instance, time-based experiments were designed to follow the decay rate of the chemiluminescence traces while the temperature remained constant, equal to that used in the oxygen electrode.

### 4.4. ITC Analysis

ITC experiments were carried out using an Auto-iTC200 system (MicroCal, Malvern-Panalytical, Malvern, England, UK), as described, in detail, in [48,53]. The BSA was, first, purified by size-exclusion chromatography using a Superdex 200 10/30 HR column connected to an ÄKTA-purifier system (GE Healthcare Bio-Sciences AB, Uppsala, Sweden). The Superdex 200 10/30 HR column was calibrated with the standard proteins of the gel filtration high molecular weight (HMW) kit supplied by GE Healthcare. A 12.5 μM solution of monomeric BSA in PBS pH 7.4 was titrated at 25 °C with a 300 μM SOCL solution. Sets of 19 injections of 2 μL were performed. The resulting heats were integrated and normalized by the amount of ligand added per injection. The heat of SOCL dilution was accounted for by introducing an additional adjustable parameter in the analysis. Further details about the ITC analysis were given in Section 2.

## 5. Conclusions

Of particular interest was the boost in the chemiluminescence intensity of the SOCL phenoxy-dioxetane after the binding of SOCL to BSA. Certain factors, such as local viscosity and the conformational restrictions of the SOCL phenoxy-dioxetane in the two binding sites of BSA, are proposed to favorably contribute to the CIEEL process during the decomposition of its phenoxy-dioxetane. Hydrophobic interactions, which do not require a high degree of specificity, were the main driving forces for the binding affinity of SOCL for BSA. This suggests that the emissive enhancement of the SOCL phenoxy-dioxetane should not be a surprise in living cells or cell compartments where proteins occupy a large fraction of the dry matter. This led us to conclude that the hydrophobic interactions of SOCL or SOCL-CPP with proteins should be taken into account when using them to detect and image endogenously- or artificially-photosensitized ^1^O_2_ in living cells.

## Figures and Tables

**Table 1:** List of the best-fitted values for the binding energetics parameters between the Singlet Oxygen Chemiluminescence probe (SOCL) and bovine serum albumin (BSA), as determined by isothermal titration calorimetry (ITC).

*K*_a_ (M^−1^)	*K*_d_ (μM)	Δ*H* (kJ mol^−1^)	n
(5.9 ± 0.9) × 10^5^	1.7 ± 0.3	7.9 ± 1.2	2.1 ± 0.1

## Supplementary Materials

The following are available online at https://www.mdpi.com/1420-3049/24/13/2422/s1, Figure S1: Time-dependence of the chemiluminescence traces of the SOCL phenoxy-dioxetane derivative after the photo-oxygenation of SOCL in equilibrium with an atmosphere of pure molecular oxygen.
