# Supplementary material for: Emissive Enhancement of the Singlet Oxygen Chemiluminescence Probe after Binding to Bovine Serum Albumin
Source: Molecules. 2019 Jul 1;24(13):2422. doi: 10.3390/molecules24132422 (PMC6651777; doi:10.3390/molecules24132422)
Supplement: Supplementary file 1 [file molecules-24-02422-s001.pdf]

SUPPLEMENTARY MATERIAL

Emissive enhancement of the Singlet Oxygen Chemiluminescence probe after binding to bovine serum albumin

Jon Miranda-Apodaca[1], Nir Hananya[2], Adrián Velázquez-Campoy[3], Doron Shabat[2] and Juan B. Arellano[1,*]

[1]Departamento de Estrés Abiótico, Instituto de Recursos Naturales y Agrobiología de Salamanca (IRNASA-CSIC), Salamanca 37008, Spain; jon.miranda@irnasa.csic.es (J.M.A)

[2]School of Chemistry, Faculty of Exact Sciences, Tel Aviv University, Tel Aviv 69978, Israel; hananyanir@gmail.com (N.H.); chdoron@post.tau.ac.il (D.S.)

[3]Institute of Biocomputation and Physics of Complex Systems (BIFI), Joint Units IQFR-CSIC-BIFI, and GBsC-CSIC-BIFI, Universidad de Zaragoza, Zaragoza 50018, Spain; Department of Biochemistry and Molecular and Cell Biology, Universidad de Zaragoza, Zaragoza 50009, Spain; Aragon Institute for Health Research (IIS-Aragon), Zaragoza 50009, Spain; Biomedical Research Networking Center in Digestive and Hepatic Diseases (CIBERehd), Madrid, Spain; Fundacion ARAID, Government of Aragon, Zaragoza 50018, Spain; adrianvc@unizar.es (A.V.-C.)

[*]Correspondence: juan.arellano@irnasa.csic.es; Tel.: +34 923 219 606

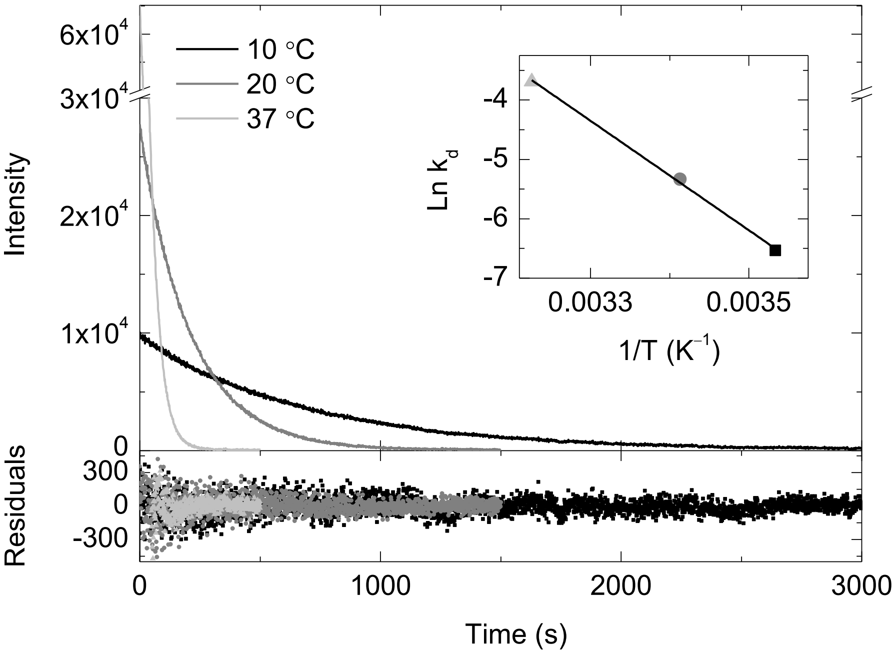

Figure S1. Time dependence of the chemiluminescence traces of the SOCL phenoxy-dioxetane derivative after the photooxygenation of SOCL with $^1O_2$ in PBS pH 7.4 at three different temperatures. Before monitoring the chemiluminescence traces, the reaction mixtures were set in the oxygen electrode chamber in equilibrium with an atmosphere of 100% O_2. 2 μM MB was present as $^1O_2$ photosensitizer and 0.5 mM SOCL as $^1O_2$ quencher. The reaction mixtures were illuminated with a red LED source with an intensity of 2 mE m^{-2} s^{-1} for 2 min. Samples were taken from the oxygen electrode chamber after illumination and placed in a 1-cm fluorescence cuvette pre-incubated each time at the temperature used in the oxygen electrode chamber. The inset shows the dependence of the decomposition rate constant of the SOCL phenoxy-dioxetane derivative on temperature using the Arrhenius equation. The data was fitted to a mono-exponential equation, $I_{515nm} = \alpha_0 + \alpha_1 \exp(-t/\tau_1)$; also shown are the corresponding residuals for each temperature experiment. The standard error for the decay rate constants is < 5%.
